# Hydrogen-bonded diketopyrrolopyrrole (DPP) pigments as organic semiconductors

**DOI:** 10.1016/j.orgel.2014.09.038

**Published:** 2014-12

**Authors:** Eric Daniel Głowacki, Halime Coskun, Martin A. Blood-Forsythe, Uwe Monkowius, Lucia Leonat, Marek Grzybowski, Daniel Gryko, Matthew Schuette White, Alán Aspuru-Guzik, Niyazi Serdar Sariciftci

**Affiliations:** a Linz Institute for Organic Solar Cells (LIOS), Physical Chemistry, Johannes Kepler University, Linz, Austria; b Department of Chemistry and Chemical Biology, Harvard University, Cambridge, USA; c Institute of Inorganic Chemistry, Johannes Kepler University, Linz, Austria; d Institute of Organic Chemistry, Polish Academy of Sciences, Warsaw, Poland

**Keywords:** Organic pigments, Organic field-effect transistors, Diketopyrrolopyrrole, Hydrogen-bonding, DFT calculations

## Abstract

Diketopyrrolopyrroles (DPPs) have recently gained attention as building-blocks for organic semiconducting polymers and small molecules, however the semiconducting properties of their hydrogen-bonded (H-bonded) pigment forms have not been explored. Herein we report on the performance of three archetypical H-bonded DPP pigments, which show ambipolar carrier mobilities in the range 0.01–0.06 cm^2^/V s in organic field-effect transistors. Their semiconducting properties are correlated with crystal structure, where an H-bonded crystal lattice supports close and relatively cofacial π–π stacking. To better understand transport in these systems, density functional theory calculations were carried out, indicating theoretical maximum ambipolar mobility values of ∼0.3 cm^2^/V s. Based on these experimental and theoretical results, H-bonded DPPs represent a viable alternative to more established DPP-containing polymers and small molecules where H-bonding is blocked by N-alkylation.

## Highlights

• H-bonded diketopyrrolopyrroles (DPPs) show ambipolar mobilities ~0.01–0.06 cm^2^/V s.
• H-bonded crystal lattice supports close and relatively cofacial π–π stacking.
• DFT calculations are used to predict mobility.
• Crystal engineering H-bonded DPPs can be a viable way to obtain useful semiconductors.

## Graphical abstract

**Figure:**
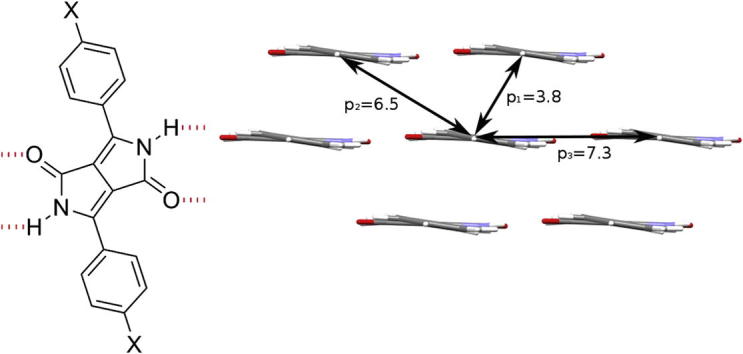

## 1 Introduction

**Fig. 1:**
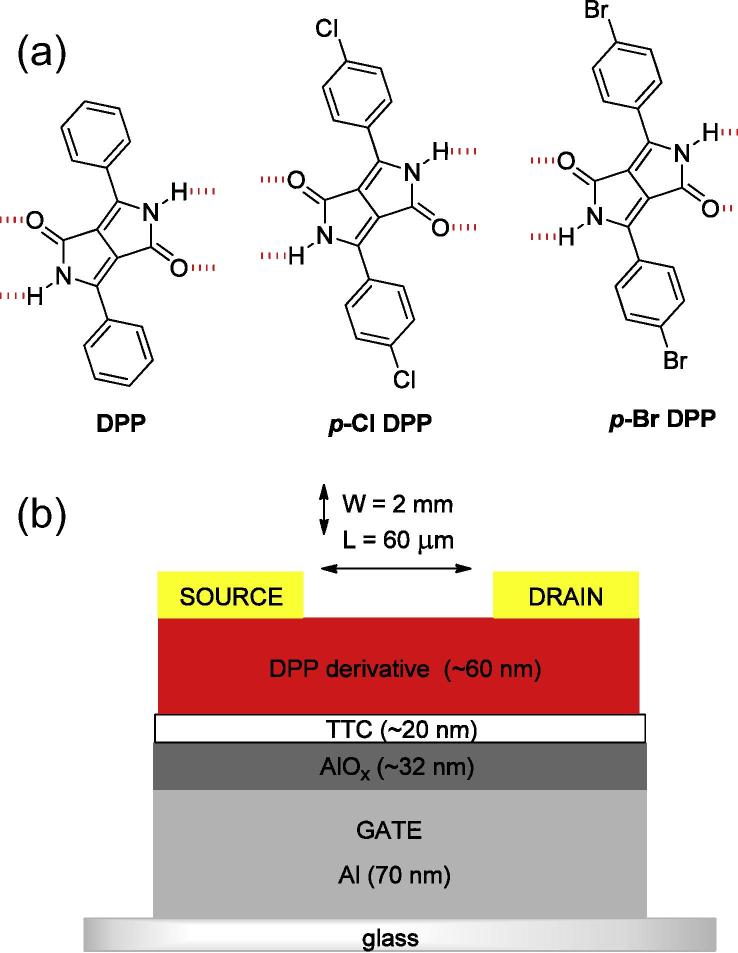
(a) Molecular structures of the three DPP derivatives used in this paper. Red dashed lines show where hydrogen bonds are formed with neighboring molecules. (b) Schematic of the OFET devices used in this study. (For interpretation of the references to colour in this figure legend, the reader is referred to the web version of this article.)

Diketopyrrolopyrroles (DPPs) are a family of high-performance industrial pigments first developed and marketed by Ciba-Geigy [1]. Like the related indigo, quinacridone, imidazolone, isoindoline, and numerous azo-pigments, many properties of DPPs originate from the interplay of intermolecular hydrogen-bonding (H-bonding; —NH—O=) and π–π stacking. This combination of intermolecular interactions leads to high crystal lattice energies, remarkable thermal stability, and excellent photostability [2], [3]. It follows that such characteristics are likewise desirable for active materials in mass-producible low-cost organic electronics. Manipulation of the H-bonding in industrial pigments is a common technique of pigment crystal engineering to achieve desired optical and mechanical properties [4], [5]. Though the concept of hydrogen-bond-mediated crystal engineering is well-established in the field of high performance organic pigments [2], [6], it has received little attention for the design of organic semiconducting materials. Promising recent demonstrations of H-bond forming perylene bisimides [7], [8], indigos, [9], [10] and quinacridone [11] motivated us to evaluate the potential of the intermolecular H-bond-forming DPPs for organic electronics. DPPs have emerged in recent years as one of the most successful building blocks in polymers, oligomers, and small molecules for high-mobility transistor materials [12] and photovoltaic devices [13]. Mobilities exceeding 1 cm^2^/V s have been reported for various DPP-based polymers and small molecules. In these applications, the DPP moieties are functionalized with solubilizing alkyl groups at the lactam nitrogens, thus eliminating the effects of H-bonding. To our knowledge, two reports of DPPs retaining free NH groups available for H-bonding exist. Yanigasawa et al. measured the mobility of simple unfunctionalized DPP (Fig. 1a) in OFETs, finding limited hole transport with a mobility of ∼1 × 10^−5 ^cm^2^/V s [14]. More recently, Lee et al. demonstrated that DPP-based polymers with some free NH groups show ambipolar mobility in the 10^−3^–10^−2^ cm^2^/V s range [15]. Many researchers in the field of organic semiconducting materials have concluded that amine and carbonyl groups should be avoided in the design of semiconducting molecules, as they can be seen to interrupt conjugation [16], [17]. However, in the case of small molecules, charge transport through π–π stacked molecules is usually the mobility-limiting process, therefore maximizing the charge transfer integrals between neighboring molecules can lead to the largest practical increases in mobility. In this respect H-bond mediated crystal engineering can be used to improve performance. From recent experience with H-bonded semiconducting molecules such as indigos, we have found that low surface-energy dielectrics are absolutely crucial to promote high mobility in H-bonded small molecule devices [18]. By employing anodically-grown AlO*_x_* passivated with tetratetracontane (C_44_H_90_, TTC) as a composite low surface-energy dielectric, we have been successful in measuring ambipolar transport in three simple DPPs (Fig. 1a): diphenyl–DPP (Pigment Red 255), di(*p*-chlorophenyl)–DPP (Pigment Red 254), and di(*p*-bromophenyl)–DPP (a frequent building block for DPP-containing polymers). It is worth mentioning here that DPP and the chlorinated DPP are both commercial products that can be purchased, in pure form, for <$0.5 g^–1^. Both pigments are widely used in robust outdoor paints and in LCD color filters, among many other applications. Pigment Red 254 is perhaps most famously known as the “Ferrari Red” used in automobile coatings. Herein we report measurements of the optical, electrochemical, crystalline structure, and charge transport properties of thin films of these three pigments, as well as applying theoretical density functional theory (DFT) methods to better understand charge transport and determine ways to improve and further utilize this class of materials.

## 2 Experimental

### 2.1 Materials and Methods

The three pigment-forming DPPs studied are shown in Fig. 1a. DPP was purchased from TCI, *p*-Cl DPP was obtained from Ciba-Geigy, and *p*-Br DPP was synthesized according to published procedures [19] from *p*-bromobenzonitrile by the condensation of *p*-bromobenzonitrile with diisopropyl succinate in the presence of sodium *tert*–amylate as base. All three compounds were purified by repeated temperature gradient sublimation at a base pressure of <1 × 10^−6^ mbar. Single crystals of *p*-Br DPP were grown using the physical vapor transport method inside of a borosilicate glass tube at a pressure of 1 atm, under N_2_ gas with a flow rate of 1 L min^−1^. UV–Vis (Perkin Elmer Lambda 1050) and photoluminescence measurements (PhotoMed PTI) were carried out on dilute (10–200 μM) solutions of the DPP derivatives in DMSO and on evaporated thin films deposited on glass substrates. Cyclic voltammetry was measured using evaporated thin films of the DPPs on ITO-coated glass (working electrode) with platinum as the counter electrode, and Ag/AgCl as a pseudo-reference electrode. The ferrocene/ferrocenium redox couple was used as an external standard. The values of the Fc/Fc^+^ vs. NHE and NHE vs. vacuum levels used in this work were 0.64 V and −4.75 V, respectively [20]. The electrolyte solution was 0.1 M tetrabutylammonium hexafluorophosphate in acetonitrile. Single-crystal X-ray structure determination was carried out using a Bruker Smart X2S diffractometer with a Molybdenum source, *λ* = 0.71073 Å. Structures were solved using direct methods (SHELXS-97 software package) and refined by full-matrix least-squares F^2^ (SHELXL-97). The H atom positions were calculated geometrically, and a riding model was applied in the refinement process. The structure of *p*-Br DPP is deposited under CCDC 958818. Crystal structure data were evaluated using the Mercury software package. Mercury was also used to predict powder spectra.

### 2.2 Fabrication and measurement of OFET devices

OFET devices were fabricated using a bottom-gate/top-contact configuration. Aluminum gate electrodes were evaporated onto clean glass substrates with a rate of ∼10 nm/s to a total thickness of ∼100 nm. An anodic amorphous aluminum oxide (AlO*_x_*) layer was then grown by the potentiostatic method [21] using an anodization voltage of 20 V in a buffered citric acid/sodium citrate 0.1 M electrolyte solution. The oxide growth factor was approximately 1.6 nm V^−1^, [22] giving a total oxide thickness of ∼32 nm. The AlO*_x_* layer was then passivated by evaporating a thin (20 nm) layer of TTC,[23], [24] giving a low surface energy and low trap density composite inorganic/organic gate dielectric with a capacitance of 20 nF/cm^2^. The low surface energy TTC layer is integral for the favorable growth of other H-bonded small molecules such as indigos [18]. The semiconducting DPP layer was then evaporated at a vacuum base pressure of 1 × 10^−6^ mbar using a rate of 0.1–0.2 Å/s, to a final thickness of 60 nm. Source and drain contacts of gold (enhanced p-type operation) or aluminum (enhanced n-type operation) were then evaporated through a shadow mask, giving a channel length of 60 μm, and a width of 2 mm. OFET properties were then measured using an Agilent B1500A parameter analyzer in N_2_ atmosphere.

## 3 Results and discussions

### 3.1 Optical and electrochemical properties

**Fig. 2:**
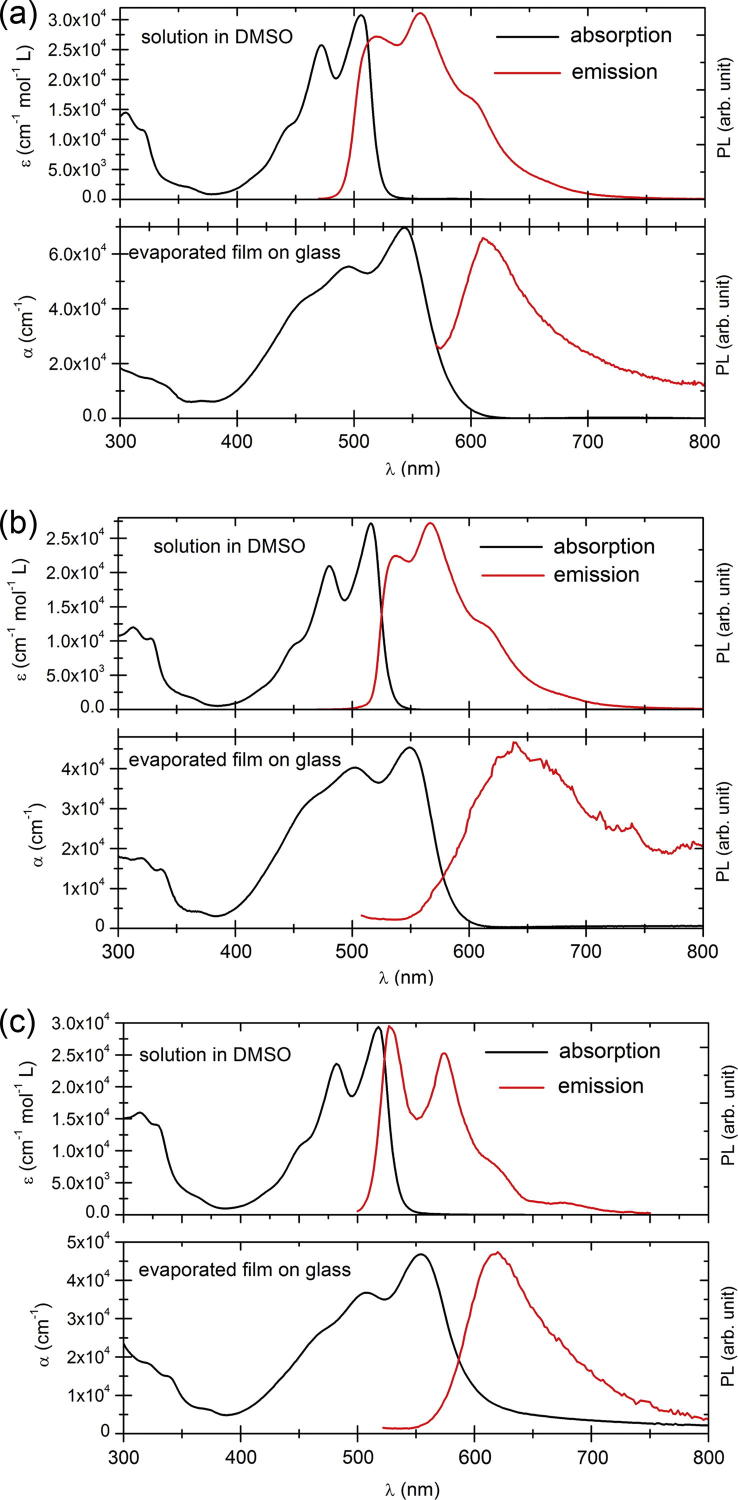
UV–Vis absorption and photoluminescence spectra of (a) DPP, (b) *p*-Cl DPP, (c) *p*-Br DPP; in DMSO solution and in thin film.

The optical absorption and photoluminescence of dilute solutions and evaporated thin films of DPP and *p*-Cl DPP and *p*-Br DPP were measured (Fig. 2). From the absorption onset in thin films the optical band gap is estimated to be 2.1 eV for all three compounds (Table 1). The absorption spectra show a pronounced bathochromic shift of about 50 nm when comparing DPPs in solution to those aggregated in the solid state. Such large shifts are indicative of both the degree of excitonic coupling between neighboring molecules [3] and the role of intermolecular H-bonding in changing the electron densities on the carbonyl oxygen and nitrogen atoms participating in the H-bond formation [25]. Photoluminescence (PL) peaks are similarly red-shifted when comparing solutions to solid state, with the well-resolved vibronic replicas visible in solution appearing as broad peaks in thin films. The PL of thin films was found to be very weak, consistent with the observation of H-bonded pigments in the solid state undergoing proton-mediated internal conversion processes [2], [26].

**Table 1:** Experimental values for semiconducting properties of the DPP pigments.

Material	Band gap (eV) CV estimate	Band gap (eV) optical	HOMO (eV)	LUMO (eV)	Exp. mobility (cm^2^/V s)
DPP	2.1	2.1	−5.4	−3.3	*μ*_e_ = 0.01 *μ*_h_ = 0.01
*p*-Cl-DPP	2.8	2.1	−6.0	−3.2	*μ*_e_ = 0.03 *μ*_h_ = 0.01
*p*-Br-DPP	2.7	2.1	−6.1	−3.4	*μ*_e_ = 0.06 *μ*_h_ = 0.02

**Fig. 3:**
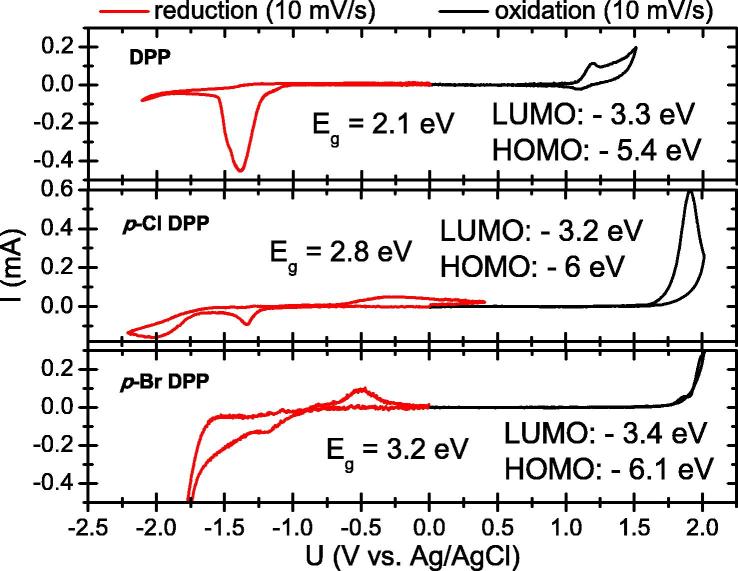
Cyclic voltammetry scans for the three DPP compounds.

In order to evaluate the possible polarity of transport in the three DPP derivatives, as well as estimate the values for the HOMO and LUMO energy levels, we performed cyclic voltammetry measurements on thin films evaporated on ITO. All three DPPs were found to afford both reduction and oxidation peaks, with quasi-reversible oxidation visible only in the case of DPP (Fig. 3). There are, however, re-oxidation and re-reduction processes visible with a significantly lower current value than the initial reduction and oxidation due to the solubility of the reduced and oxidized forms in the electrolyte and dissolution of the thin film under testing. Due to this phenomenon, evaluating reversibility *y* is problematic. Although optical band gaps for all three materials were found to be identical, the electrochemical HOMO and LUMO values, as well as band gaps, varied between the molecules. These values are compared in Table 1. Unsubstituted DPP was found to have the smallest electrochemical band gap, 2.1 eV, and also the lowest-lying LUMO level. In proceeding from unsubstituted to —Cl and —Br, the trend is decreasing HOMO energy, and thus widening band gap. The presence of electronegative halogens intuitively is expected to make the molecules more difficult to oxidize.

### 3.2 Field-effect transistor devices

**Fig. 4:**
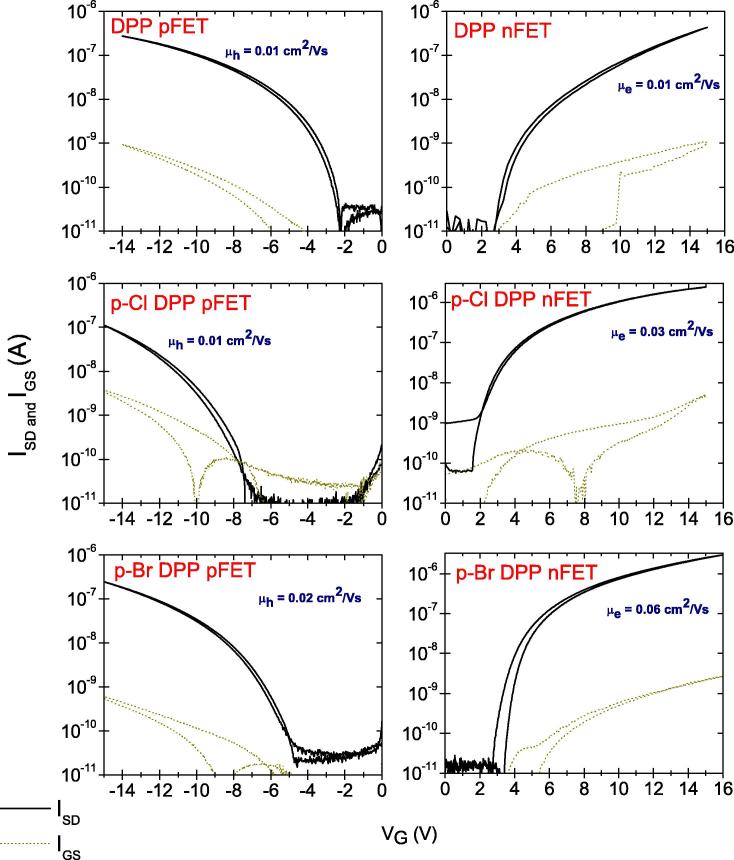
Transfer curves showing source–drain current (black traces), and gate leakage current (olive). Hole-enhanced devices shown in the column on the left were fabricated with Au source–drain electrodes, *V*_SD_ = −10 V in all cases. Electron-enhanced devices with Al source–drain electrodes are shown on the right, with *V*_SD_ = 10 V.

**Fig. 5:**
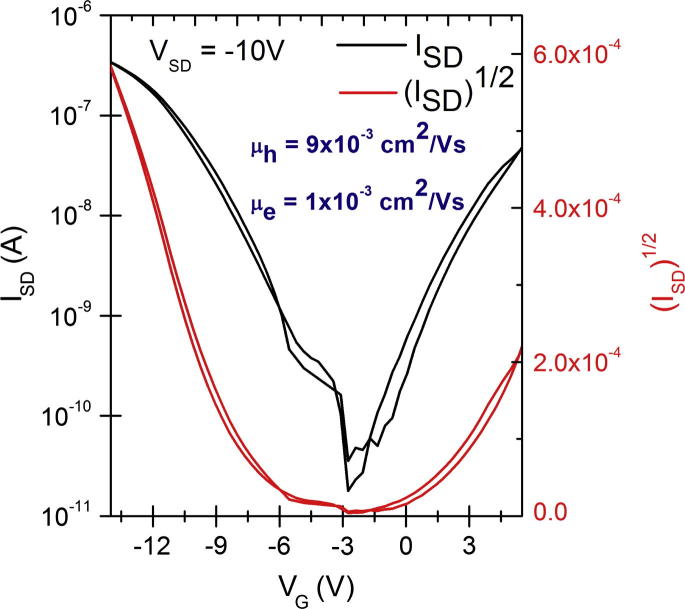
Ambipolar transfer curve for a DPP transistor with silver source–drain electrodes.

OFET devices of identical structure were fabricated to compare the charge carrier mobility of DPP and its derivatives. In order to provide a trap-free dielectric interface the aliphatic oligoethylene TTC was chosen to passivate the AlO*_x_* surface. All three materials were found to support both electron and hole transport, as can be rationalized from the observed electrochemical behavior where both oxidation and reduction processes occurred, and further by the calculated reorganization energies described in section 3.4. The measured mobility values are shown in Table 1. In order to measure hole-enhanced transport, Au was selected as a source–drain contact metal, while for electron-enhanced transport Al was used (Transfer curves shown in Fig. 4). Overall, *p*-Br DPP was found to have the highest mobility for both carriers. In all cases, hysteresis was minimal. Only n-channel devices with *p*-Cl DPP were found to have a clockwise hysteresis resulting in a slightly higher off-current. We were able to observe ambipolar FET characteristics in the case of DPP by using silver as a source–drain contact metal (Fig. 5). Since the injection in this case is not optimal for either carrier, the overall calculated mobility for electrons and holes was lower than in the more optimized unipolar devices. In the case of halogenated DPPs ambipolar FET characteristics were not observed due to the significantly higher band gap of these two derivatives.

### 3.3 Crystalline structure and thin film morphology

**Fig. 6:**
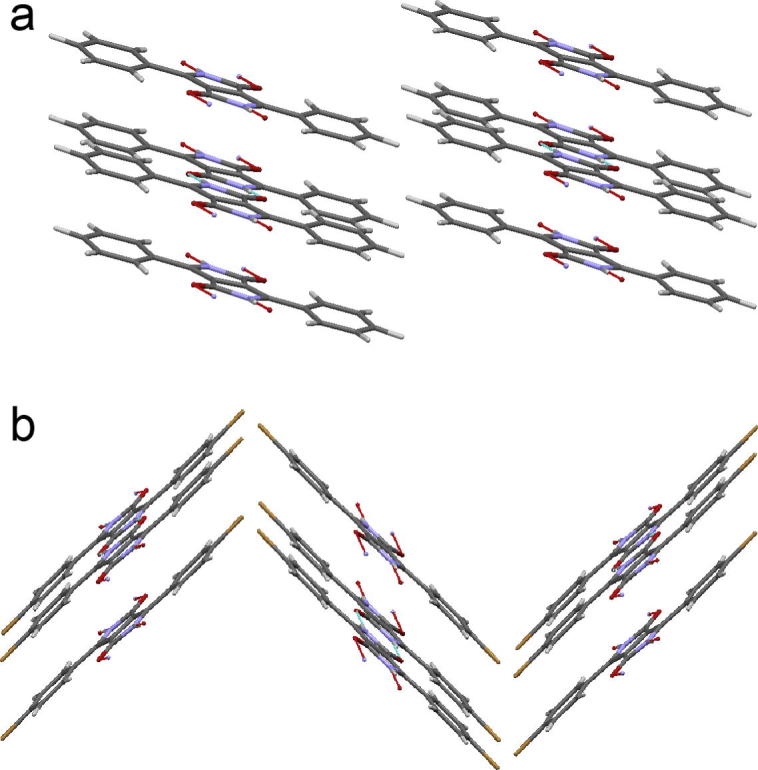
Crystal structures of DPPs. The red lines indicate H-bonds. (a) Structure of DPP, viewed down the [1], [2], [3], [4], [5], [6], [7], [8], [9], [10], [11], [12] plane. The linear-chain H-bonding pattern is characteristic of all three DPPs, but only in the case of DPP are the chains parallel to one another. (b) *p*-Br DPP viewed down the [0 2 0] plane. The staggered head-to-tail arrangement of H-bonded chains is shared by both *p*-Cl DPP and *p*-Br DPP. (For interpretation of the references to colour in this figure legend, the reader is referred to the web version of this article.)

**Fig. 7:**
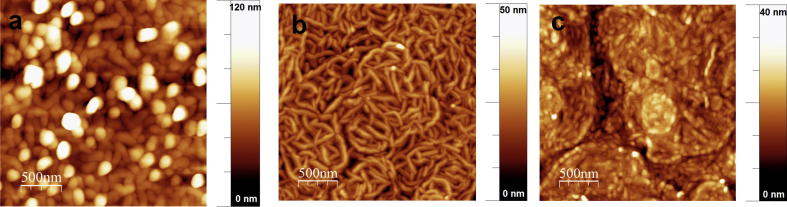
AFM micrographs of thin films of (a) DPP, (b) *p*-Cl DPP, and (c) *p*-Br DPP, all deposited on AlO*_x_*/TTC gate dielectrics.

Crystal structures for various DPP pigments were reported by Mizuguchi and co-workers from Ciba-Geigy around 1990 [27], [28]. All H-bonded DPP pigments to-date have been shown to crystallize in linear H-bonded chains, where each molecule has head-to-tail double H-bonds to two neighbors. In DPP, the H-bonded chains are all parallel to one another, running parallel to the [1], [2], [3], [4], [5], [6], [7], [8], [9], [10], [11], [12] plane, while brick wall-pattern π–π stacking is visible perpendicular to the [1], [2], [3], [4], [5], [6], [7], [8], [9], [10], [11], [12] plane (Fig. 6a). The H-bond length in all DPP pigments here, 1.7–1.8 Å, is very short in comparison to other pigment-forming molecules. For example, quinacridones have ∼2 Å, and indigos 2.1–2.8 Å. The *para*-halogenated DPP derivatives also have a linear chain H-bonding motif, but the chains are staggered relative to each other in such a way that two linear H-bonded chains run along the [0 0 1] plane, and another two chains run along the [0 0 2] plane, tilted 89° clock-wise with respect to the first chain. The crystal structure we obtained for *p*-Br DPP is qualitatively very similar to the known *p*-Cl DPP structure, and can be described as pseudo brick-wall π–π stacking (Fig. 6b). The interplanar spacing and intermolecular centroid–centroid distances for π–π stacking for all three materials are shown in Table 2. From the point of view of crystalline packing in the π-stacking and H-bonding plane the three materials are all very similar and no differences immediately visible from their relative structures suggest discrepancies in charge transport behavior. However, one would expect a transport anisotropy in all cases, with charge transport favorably occurring along the π–π stacking direction, i.e., perpendicular to the H-bonding direction. Thus orientation of the π–π stacking parallel to the gate dielectric is critical for optimal transport in transistors [29], [30]. It was reported that hydrophobic substrates are necessary to ensure such a stacking direction, as the hydrophobic van der Waals contacts of the H-bonded pigment molecules favorably interact with such surfaces. We found that TTC/AlO*_x_* is an ideal dielectric in this respect for H-bonded pigments, including the DPPs. Out-of-plane *ω*–2*θ* XRD shows only peaks from [0 0 l] planes ([0 k 0] in case of *p*-Cl DPP), showing that indeed the molecules are in a “standing” orientation on the TTC/AlO*_x_* (Fig. S1). Diffraction from other planes is very weak or absent, indicating strong anisotropy of growth. The position of the [0 0 l] peaks, as well as the other, weaker, diffraction peaks, is consistent with expected diffraction angles calculated based on the single crystal structures. Thin films on glass showed no peaks, indicating little or no preferential orientation. Despite the preferential orientation occurring when DPPs are grown on TTC/AlO*_x_*, charge transport may still be limited by the polycrystalline morphology of such films. Fig. 7 shows atomic force micrographs (AFM) of the active channel region of FETs with each DPP. DPP forms crystal grains 100–200 nm in size, with clearly defined boundaries between crystallites. Crystallites of *p*-Cl DPP are of similar size but more oblong in shape. In the case of *p*-Br DPP, smaller grains (100 nm maximum) form a highly smooth film with considerably less apparent grain boundaries. Intercrystallite resistance may be a limiting factor in the mobility of such highly crystalline pigments, the smoother and more continuous morphology of *p*-Br DPP may explain the higher mobility values found experimentally for this material (see Fig. 7).

**Table 2:** Data for crystalline ordering obtained from single-crystal X-ray diffraction.

Material	Interplanar π–π distance (Å)	Intermolecular π–π (centroid–centroid) distance (Å)	N—H—O=bond length (Å)	Packing pattern	Space group
DPP	3.3 (along ***a***) 3.0 (along ***b***)	3.817 (along ***a***) 6.516 (along ***b***)	1.816	Brick-wall	P–1
*p*-Cl–DPP	3.0 (along ***a***) 3.3 (along ***c***)	5.658 (along ***a***) 5.585 (along ***c***)	1.740	Pseudo-brick-wall	P21/n
*p*-Br–DPP	3.2 (along ***b***) 3.2 (along ***a***)	5.628 (along ***b***) 5.628 (along ***a***)	1.770	Pseudo-brick-wall	C2/c

### 3.4 Theoretical calculations

We conducted density functional theory (DFT) calculations to predict the mobilities of the three DPP materials in their X-ray determined bulk crystal structures. These calculations provide an estimate of the intrinsic mobility in the pigment crystals, and indicate if the observed trend in mobility is related to the material itself or more to the film morphology. At room temperature, charge carriers are expected to be localized to a single or a few molecules due to strong electron–phonon coupling. In the case of localization to a single molecule, the mobility is then predicted by calculating the diffusive hopping rate *k_i_* between molecules spaced a distance *d_i_* apart. The 3D diffusion constant was estimated both through averaging over random walk simulations (to observe anisotropy) and via the analytic approximation for isotropic diffusion [31]:

$$D\;\approx \;\frac{1}{2n}\underset{i}{\sum }d_{i}^{2}k_{i}P_{i}$$

where *P_i_* = *k_i_*/∑*_i_k_i_* is the relative probability of hopping to the *i*^th^ nearest neighbor molecule, and *n* is the dimensionality. The mobility is related to the diffusion coefficient by the Einstein relation *μ* = *eD*/*k_B_T*. The Marcus theory semi-classical rate *k* for charge transfer [32], [33] (i.e. the high temperature limit) is calculated as:

$$k_{i}=t_{i}^{2}\sqrt{\frac{\pi }{\hbar^{2}k_{B}T\lambda }}e^{-\frac{{\left(\lambda +\Delta \varepsilon_{i}\right)}^{2}}{4\lambda k_{B}T}}$$

where λ is the internal reorganization energy calculated as the geometric relaxation energy of the charged species, *t* is the intermolecular transfer integral calculated using the projective method [34], and Δ*ɛ* is the difference in site energies. All calculations were performed at the DFT level with two functionals: B3LYP [35], [36] and PW6B95 [37]. We employed Grimme’s atom-pairwise dispersion correction with Becke–Johnson damping (D3BJ)[38] and the Ahlrichs def2-TZVP basis set [39]. TZP quality basis sets have previously been shown to yield reliable results for transfer integrals in pentacene [40] and ethylene [41] dimers. The reorganization energies were calculated using a smaller def2-SVPD basis set [42]. All calculations were performed employing the ORCA v3 software package [43].

The calculation results are presented in Table 3, where the stated mobilities correspond to the analytic approximation to 3D isotropic diffusion. The isotropic result is chosen for comparison with experiment due to the polycrystalline nature of the thin film transistors. However, there is strong mobility anisotropy, which is discussed in detail in the Supplemental Information. Calculated HOMO–LUMO gaps agree with the observation of a similar bandgap for all three pigments seen in optical experiments. The predicted electron mobilities agree within a factor of 2 to 9 with the experimental values presented in Table 1 and correctly predict the trend of Cl and Br substitution increasing the mobility. The calculated hole mobilities are in greater disagreement, off by up to a factor of 17. All calculations predicted higher hole mobility than electron mobility, primarily due to a single strong π–π transport pathway. It is unsurprising that the calculated mobilities are as much as an order of magnitude larger than the experimental values because this model does not incorporate any disorder or renormalization due to electron–phonon coupling.

**Table 3:** Theoretical values for semiconducting properties of DPP pigments. Band gaps and mobilities were calculated with density functional theory using the B3LYP (PW6B95) functional with D3BJ dispersion correction and def2-TZVP basis set. Reorganization energies were calculated with the same functionals but using the smaller def2-SVPD basis set.

Material	Band gap (eV)	HOMO (eV)	LUMO (eV)	Reorganization energy (meV)	Calc. mobility (cm^2^/V s)
DPP	1.6 (1.6)	−3.3 (−3.2)	−1.6 (−1.5)	*λ*_e_ = 184 (241) *λ*_h_ = 317 (353)	*μ*_e_ = 0.09 (0.04) *μ*_h_ = 0.08 (0.05)
*p*-Cl–DPP	1.4 (1.5)	−3.0 (−2.9)	−1.6 (−1.5)	*λ*_e_ = 182 (192) *λ*_h_ = 328 (354)	*μ*_e_ = 0.09 (0.08) *μ*_h_ = 0.17 (0.12)
*p*-Br–DPP	1.6 (1.6)	−2.8 (−2.6)	−1.2 (−1.0)	*λ*_e_ = 175 (186) *λ*_h_ = 316 (344)	*μ*_e_ = 0.12 (0.10) *μ*_h_ = 0.29 (0.20)

**Fig. 8:**
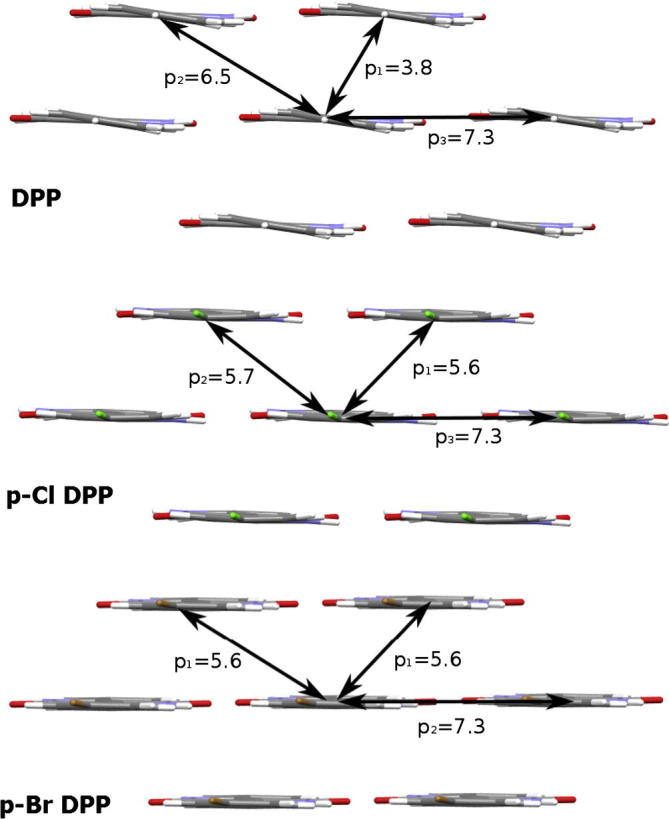
Dominant charge hopping pathways for DPPs considered in density functional theory transport calculations. Distances between centroids are in units of Å. The calculated charge transfer integrals show that both pathways along π–π stacking and H-bonding contribute significantly to charge transport.

By examining the charge transfer integrals we can determine which pathways, or nearest-neighbor pairs, are contributing most strongly to the predicted mobility (see Supplementary Information for tables of the transfer integrals). One feature of transfer integral predictions is that they are extremely sensitive to both the overlap of the frontier orbitals of the dimer of interest and also to the relative phase of those orbitals. The nearest-neighbor pathways contributing most significantly to charge transport are displayed for all three DPPs in Fig. 8. The structural and electronic perturbations introduced by Cl and Br substitutions alter which pathway is dominant in the mobility expression. For instance, the electron mobility of DPP is dominated by the H-bond pathway, while *p*-Cl DPP and *p*-Br DPP show stronger electron transport in the π–π pathways. An important observation is that a substantial contribution (12–70%) is made by pathways along the H-bonding direction in all three compounds. The edge-to-edge contacts in molecular semiconductors normally have very low transfer integrals and contribute negligibly to transport. In the case of DPPs, however, these values are considerably higher. This is potentially significant, as not only π–π stacking contacts are thus expected to lead to conduction, but also H-bonding contacts. This implies that the prevailing understanding concerning orientation of π–π stacking domains, as described above in Section 3.3, may require reconsideration with respect to H-bonded systems. Therefore, in the design of future H-bonded derivatives of DPP, increasing H-bonding interactions may be a favorable approach. Additional considerations on crystal engineering DPPs are presented in the Supplementary information.

## 4 Conclusions

Our results show that H-bonded DPP pigments can function as semiconductors in their own right, without incorporation into more complex derivative materials. The combination of X-ray crystal structure data and theoretical DFT treatment allows detailed understanding of how charge is transported in such crystal systems, where closer π–π stacking distance corresponds to higher mobility. Interestingly, according to the calculated charge transfer integrals, the H-bonded intermolecular contacts can also contribute significantly to transport in the crystal. Though the mobility values remain modest in comparison with more optimized DPP-containing polymeric systems, in the 10^−2^–10^−1^ cm^2^/V s range, these results suggest the possibility of expanding research in the direction of H-bonded crystal engineered materials. Routes to improve mobility in DPP derivatives may be (1) to decrease the internal reorganization energy for electrons or holes by adding electron-withdrawing or electron-donating substituents, respectively; or (2) increase the charge transfer integral contribution from the H-bonding direction by adding more H-bonding functional groups. These results can help to guide future synthetic efforts and predictive computational models to make crystal-engineered DPPs with higher performance.
