# Supplementary material for: Hydrogen-bonded diketopyrrolopyrrole (DPP) pigments as organic semiconductors
Source: Org Electron. 2014 Dec;15(12):3521–8. doi: 10.1016/j.orgel.2014.09.038 (PMC4307998; doi:10.1016/j.orgel.2014.09.038)
Supplement: Supplementary data 1 [file mmc1.docx]

**Supplemental Information for:**

**Hydrogen-bonded diketopyrrolopyrrole (DPP) pigments as organic semiconductors**

Eric Daniel Głowacki,^1^* Halime Coskun,^1^ Martin A. Blood-Forsythe,^2^ Uwe Monkowius,^3^ Lucia Leonat,^1^ Marek Grzybowski,^4^ Daniel Gryko,^4^ Matthew Schuette White,^1^ Alán Aspuru-Guzik,^2^ Niyazi Serdar Sariciftci^1^

*^1^Linz Institute for Organic Solar Cells (LIOS), Physical Chemistry, Johannes Kepler University, Linz, Austria*

*^2^Department of Chemistry and Chemical Biology, Harvard University, Cambridge, USA*

*^3^Institute of Inorganic Chemistry, Johannes Kepler University, Linz, Austria*

*^4^Institute of Organic Chemistry, Polish Academy of Sciences, Warsaw, Poland*

***Thin film XRD***





**Figure S1.** Out-of-plane ω-2θ XRD for DPP evaporated on TTC/AlO_x_ compared with powder spectrum calculated based on known single-crystal structure. The [001] diffraction is very prominent, indicating that the molecules grow in an anisotropic fashion, such that the c-axis of the DPP unit cell is perpendicular to the substrate.

***Single Crystal X-Ray Diffraction***

**Table S1.** Crystal structure data for *p*-Br DPP.

| Compound | *p*-Br DPP |
| --- | --- |
| Formula | [C_18_H_10_Br_2_N_2_O_2_](file:///C:\Users\AK111098\Dropbox\Diketopyrrolopyrrole%20device%20paper%202013\I%20_chemical_formula_moiety) |
| Molecular weight [g mol^-1^] | [446.10](file:///C:\Dropbox\Uwe\manuskript\I%20_chemical_formula_weight) |
| Size [mm] | [0.05](file:///C:\Dropbox\Uwe\manuskript\I%20_exptl_crystal_size_max)×[0.05](file:///C:\Dropbox\Uwe\manuskript\I%20_exptl_crystal_size_mid)×[1.00](file:///C:\Dropbox\Uwe\manuskript\I%20_exptl_crystal_size_min) |
| Crystal system | [monoclini](file:///C:\Dropbox\Uwe\manuskript\I%20_space_group_crystal_system)c |
| Space group | *C*2/*c* |
| a [Å] | [7.297(3)](file:///C:\Dropbox\Uwe\manuskript\I%20_cell_length_a) |
| b [Å] | [8.571(3)](file:///C:\Dropbox\Uwe\manuskript\I%20_cell_length_b) |
| c [Å] | [23.89(1)](file:///C:\Dropbox\Uwe\manuskript\I%20_cell_length_c) |
| β [°] | 98.68(1) |
| V [Å^3^] | 1477.0(9) |
| ρ_calcd_ [g cm^-3^] | 2.006 |
| Z | 4 |
| µ [mm^-1^] | [5.50](file:///C:\Dropbox\Uwe\manuskript\I%20_exptl_absorpt_coefficient_mu) |
| T [K] | [200](file:///C:\Dropbox\Uwe\manuskript\I%20_cell_measurement_temperature) |
| θ [°] | 3.5 – 22.9 |
| Measured reflections | 7490 |
| Independent reflections | [1820](file:///C:\Dropbox\Uwe\manuskript\I%20_reflns_number_total) |
| Reflections with *I* > 2σ(*I*) | 1048 |
| Restraints/ refined parameters | 0/97 |
| Absorption correction | multi-scan |
| T_min_, T_max_ | [0.29](file:///C:\Dropbox\Uwe\manuskript\I%20_exptl_absorpt_correction_T_min), [0.77](file:///C:\Dropbox\Uwe\manuskript\I%20_exptl_absorpt_correction_T_max) |
| ρ_max/min_ [e Å^-3^] | [3.18](file:///C:\Dropbox\Uwe\manuskript\I%20_refine_diff_density_max)/[-2.7](file:///C:\Dropbox\Uwe\manuskript\I%20_refine_diff_density_min)5 |
| R_1_ [F^2^ ≥ 2σ(F^2^)] | 0.165 |
| wR_2_(F^2^) | 0.455 |
| CCDC number | 958818 |

***Theoretical Considerations, continued***:

**Mobility anisotropy:**

The dominant contribution to hopping transport in hydrogen-bonded DPP pigments occurs in the π-stacking and H-bonding plane where the greatest overlap between frontier orbitals of neighboring molecules occurs. The packing arrangement of this plane is very similar across all three pigments even though DPP forms a more linear head-to-tail arrangement than p-Cl DPP and p-Br DPP. **Figure S2** shows the hopping pathways that are the dominant transport directions in DPP. Paths 1 and 2 are π-π interactions and path 3 is a hydrogen-bond interaction. The same labeling scheme is used for the pathways in the transport plane of p-Cl DPP and p-Br DPP.

**Table S3** presents the contributions of each hopping pathway to the calculated transport properties for DPP. Likewise, **Table S4** and **Table S5** present the same breakdown for p-Cl DPP and p-Br DPP. The contribution of each pathway to the 3D isotropic diffusive mobility is included as:

$$\mu_{i}=\frac{eD_{i}}{k_{B}T}=\frac{e}{6k_{B}T} {d_{i}}^{2}k_{i}P_{i}$$

It is particularly unusual that the dominant electron transport pathway for DPP occurs along a hydrogen-bond pathway rather than a π-stacking interaction. In p-Cl DPP and p-Br DPP the π-π pathways are more dominant, but the hydrogen-bond interactions are still highly probable hopping pathways.

To visualize the mobility anisotropy we have performed a random walk simulation and then computed the directionally dependent diffusion constant:

$$D_{1D}^{random walk}\left( \theta,\phi\right)=\frac{1}{2}\lim_{N\to\infty} \frac{\left\langle{(r_{N}\cdot\hat{n})}^{2}k \right\rangle}{N}$$

where $r_{N}$ is the displacement vector after N steps, $\hat{n}$ is a unit vector in the $\left( \theta,\phi\right)$ direction, and $k$ is the charge transfer rate (which converts from the number of steps in a random walk to inverse time as usually appears in a diffusion constant). In practice this quantity is computed at large N averaging over many instances of the random walk until convergence is reached. Note that the prefactor in the above equation is 1/2 rather than 1/6 because the projection onto $\hat{n}$ means that this is a 1D diffusion constant. **Figure S3** (**Figure S4**) shows the anisotropic hole (electron) mobility computed from $D_{1D}^{random walk}\left( \theta,\phi\right)$. Note that since the angular projection uses a 1D diffusion constant definition, the mobilities are 3 times larger than the pathway contributions to $\mu_{3D,iso}$ displayed in **Tables S3-S4**. This dimensional scaling is required to make the spherical average of $D_{1D}^{random walk}\left( \theta,\phi\right)$ equal$D_{3D,iso}^{random walk}$. For convenience we define the X-axis along the H-bond and the Y-axis to be perpendicular to the molecular backbone (in the direction of the π-interactions), leaving the head-to-tail contacts in the Z direction. With this definition, the “transport plane” defined by pairs 1-3 is the XY plane. As expected, hole mobility is significantly higher in the XY plane than in the head-to-tail Z directions. In **Figure S3** the tilts of the mobility curves in XY plane originate from the dominance of the p_2_ π-π pathway for p-Cl DPP and p-Br DPP and the p_1_ π-π pathway for DPP. In **Figure S4** electron transport has strong contributions from the H-bond interactions along X, but for p-Cl DPP and p-Br DPP there are also strong contributions from head-to-tail interactions, which causes the greater elongation along the Z direction.

To check for consistency with the analytic approximation for the 3D isotropic diffusion constant,

$$D_{3D,iso}\approx\frac{1}{6}\sum_{i} {d_{i}}^{2}k_{i}P_{i}$$

we compute an overall 3D isotropic diffusion constant from the random walks as:

$$D_{3D,iso}^{random walk}=\frac{1}{6}\lim_{N\to\infty} \frac{\left\langle{r_{N}}^{2}k \right\rangle}{N}$$

The results of this comparison are presented in **Table S2**; in general there is good agreement between the analytic approximation to the 3D isotropic mobility and the results of the random walk simulations. As a further sanity check we used Lebedev quadrature (order 35) to compute the spherical average of $D_{1D}^{random walk}\left( \theta,\phi\right)$ and found agreement to at least 2 decimal places with $D_{3D,iso}^{random walk}.$

**Possible strain induced modification of the π-π pathway:**

To investigate the surprisingly weak contributions of the π-π pathways to the transport in DPP relative to the H-bond pathway, we performed additional charge transfer integral calculations (using B3LYP-D3BJ/def2-TZVP) on a series of 9 additional translated dimers that interpolate between the positions of pair 1 and pair 2 in the experimental crystal structures. As shown in the insets of figures S2-S4, these pairs were translated in along the unit vector pointing from the position of pair 1 to pair 2. Translation in this manner maintained the interplanar distance. **Figure S5** shows the results of this translation for DPP. As expected, we see the transfer integral oscillate, which reflects the relative phase and overlap between the frontier orbitals. These calculations suggest that a moderate sheer in the DPP layers, as might be accomplished with strain or steric modifications, might significantly enhance transport along the π pathways. The magnitudes of π-π transfer integrals achieved by this relative displacement scan indicate that even if the thin film structure of DPP differs in the π-direction from the bulk single crystal, the H-bond pathway is still likely to make a significant contribution to transport. **Figure S6** and **Figure S7** show the same series of calculations for p-Cl DPP and p-Br DPP respectively.

**Calculation details:**

The transport properties collected in tables S2-S4 were calculated at with density functional theory using the B3LYP and PW6B95 functionals with Grimme’s atom-pairwise Becke-Johnson damping (D3BJ) dispersion correction. The dispersion correction was applied at all stages of geometry optimization and single point energy calculations. Transfer integrals and site energies were calculated using the Ahlrichs def2-TZVP basis set. The reorganization energies used to compute the transfer rates (and therefore hopping probabilities and mobilities) were computed with Rappoport’s property optimized def2-SVPD basis set. Reorganization energy calculations were repeated with the def2-TZVP basis set, but convergence difficulties with
p-Cl DPP prevented us from using the TZVP level reorganization energies for comparison of transport with all three pigments. The SVPD level reorganization energies for DPP and p-Br DPP agree within 5-10 meV of the TZVP level reorganization energies in most cases. These results are presented in **Table S6**. For the most part, the predictions of the B3LYP functional agree well with the PW6B95 results, with PW6B95 performing somewhat better against the experimental mobilities.

**Table S2.** Summary of mobilities computed using the analytic approximation and random walk simulations. µ**_3D,iso_** corresponds to the mobility calculated from the isotropic 3D diffusion constant and µ_max_ is the maximum value of the anisotropic mobility computed using $D_{1D}^{random walk}\left( \theta,\phi\right)$. Results using the B3LYP functional are shown first, followed by the PW6B95 result in parentheses.

| Compound | Analytic  µ_3D,iso_ [cm^2^/Vs] | Random Walk  µ_3D,iso_ [cm^2^/Vs] | Random Walk  µ_max_ [cm^2^/Vs] |
| --- | --- | --- | --- |
| DPP | µ_e_ = 0.09 (0.04)  µ_h_ = 0.08 (0.05) | µ_e_ = 0.08 (0.06)  µ_h_ = 0.09 (0.06) | µ_e_ = 0.21 (0.15)  µ_h_ = 0.19 (0.11) |
| p-Cl DPP | µ_e_ = 0.09 (0.08)  µ_h_ = 0.17 (0.12) | µ_e_ = 0.09 (0.09)  µ_h_ = 0.16 (0.16) | µ_e_ = 0.12 (0.13)  µ_h_ = 0.36 (0.37) |
| p-Br DPP | µ_e_ = 0.12 (0.10)  µ_h_ = 0.29 (0.20) | µ_e_ = 0.14 (0.12)  µ_h_ = 0.25 (0.18) | µ_e_ = 0.22 (0.19)  µ_h_ = 0.64 (0.45) |

**Table S3.** DFT calculated transport properties for DPP broken down into hopping pathways. P is the relative probability of hopping along the i^th^ pathway. t is the transfer integral, Δε is the site energy difference, m is the multiplicity, i.e. the number of symmetry equivalent pathways, and μ is the contribution to the mobility from that pathway. Dominant pathways are shown in bold. Results using the B3LYP functional are shown first, followed by the PW6B95 result in parentheses.

| Path | d (Å) | m | Δε (meV) | t (meV) | P | μ (cm^2^/Vs) |
| --- | --- | --- | --- | --- | --- | --- |
| 1 | 3.8 | 2 | Δε_e_ = 7.9×10^-2^ (1.5×10^-2^)  Δε_h_ = 5.6×10^-3^ (7.3×10^-3^) | t_e_ = 5.9 (-7.8)  **t_h_ = 83 (-80)** | P_e_ = 0.05 (0.08)  **P_h_ = 0.81 (0.80)** | μ_e_ = 1×10^-4^ (2×10^-4^)  **μ_h_ = 7×10^-2^ (4×10^-2^)** |
| 2 | 6.5 | 2 | Δε_e_ = -2.6 ×10^-4^ (4.2×10^-4^)  Δε_h_ = -4.2×10^-2^ (-5.7×10^-2^) | **t_e_ = -14 (12)**  t_h_ = -23 (-23) | **P_e_ = 0.26 (0.21)**  P_h_ = 0.06 (0.06) | **μ_e_ = 9×10^-3^ (3×10^-3^)**  μ_h_ = 1×10^-3^ (9×10^-4^) |
| 3 | 7.3 | 2 | Δε_e_ = -8.9×10^-3^ (-1.4×10^-2^)  Δε_h_ = 1.4×10^-2^ (1.6×10^-2^) | **t_e_ = -22 (23)**  **t_h_ = -33 (-33)** | **P_e_ = 0.69 (0.70)**  **P_h_ = 0.13 (0.14)** | **μ_e_ = 8×10^-2^ (4×10^-2^)**  **μ_h_ = 7×10^-3^ (5×10^-3^)** |
| 4 | 13.9 | 2 | Δε_e_ = -6.7×10^-3^ (-1.1×10^-2^)  Δε_h_ = -6.2×10^-3^ (-9.7×10^-2^) | t_e_ = -1.0 (-1.0)  t_h_ = -0.35 (0.39) | P_e_ = 1.4×10^-3^ (1.4×10^-3^)  P_h_ = 1.4×10^-5^ (1.9×10^-5^) | μ_e_ = 1 ×10^-6^ (6×10^-7^)  μ_h_ = 3×10^-10^ (3×10^-10^) |
| 5 | 14.7 | 2 | Δε_e_ = -5.8×10^-3^ (-7.8×10^-3^)  Δε_h_ = -1.0×10^-2^ (-1.2×10^-2^) | t_e_ =1.8 (1.8)  t_h_ = 1.8 (1.7) | P_e_ = 4.2×10^-3^ (4.3×10^-3^)  P_h_ = 3.7×10^-4^ (3.7×10^-4^) | μ_e_ = 1×10^-5^ (6×10^-6^)  μ_h_ = 2×10^-7^ (1×10^-7^) |
| 6 | 15.1 | 2 | Δε_e_ = 1.3×10^-2^ (1.5×10^-2^)  Δε_h_ = 7.0×10^-3^ (8.2×10^-3^) | t_e_ = 0.26 (-0.27)  t_h_ = -8.2×10^-2^ (-8.4×10^-2^) | P_e_ = 9.4×10^-5^ (1.0×10^-4^)  P_h_ = 7.8×10^-7^ (8.7×10^-7^) | μ_e_ = 6×10^-9^ (4×10^-9^)  μ_h_ = 1×10^-12^ (8×10^-13^) |

**Table S4.** DFT calculated transport properties for p-Cl DPP broken down into hopping pathways. P is the relative probability of hopping along the i^th^ pathway. t is the transfer integral, Δε is the site energy difference, m is the multiplicity, i.e. the number of symmetry equivalent pathways, and μ is the contribution to the mobility from that pathway. Dominant pathways are shown in bold. Results using the B3LYP functional are shown first, followed by the PW6B95 result in parentheses.

| Path | d (Å) | m | Δε (meV) | t (meV) | P | μ (cm^2^/Vs) |
| --- | --- | --- | --- | --- | --- | --- |
| 1 | 5.6 | 2 | Δε_e_ = -1.2×10^-2^ (-1.7×10^-2^)  Δε_h_ = 1.9×10^-2^ (3.0×10^-3^) | **t_e_ = -17 (-15)**  t_h_ = 16 (-15) | **P_e_ = 0.17 (0.14)**  P_h_ = 2.6×10^-2^ (2.5×10^-2^) | **μ_e_ = 6.6×10^-3^ (4.0×10^-3^)**  μ_h_ = 1.6×10^-4^ (1.0×10^-4^) |
| 2 | 5.7 | 2 | Δε_e_ = 1.4×10^-2^ (2.0×10^-2^)  Δε_h_ = 2.2×10^-4^ (-1.6×10^-3^) | **t_e_ = -23 (24)**  **t_h_ = 89 (87)** | **P_e_ = 0.34 (0.36)**  **P_h_ = 0.83 (0.83)** | **μ_e_ = 2.6×10^-2^ (2.6×10^-2^)**  **μ_h_ = 0.16 (0.12)** |
| 3 | 7.3 | 2 | Δε_e_ = 1.5×10^-2^  (2.1×10^-2^)  Δε_h_ = 9.3×10^-3^ (1.4×10^-2^) | **t_e_ = -21 (-21)**  **t_h_ = -33 (33)** | **P_e_ = 0.26 (0.27)**  **P_h_ = 0.12 (0.12)** | **μ_e_ = 2.7×10^-2^ (2.4×10^-2^)**  **μ_h_ = 5.2×10^-3^ (4.0×10^-3^)** |
| 4 | 12.1 | 2 | Δε_e_ = 30 (30)  Δε_h_ = 29 (29) | t_e_ = 2.0 (2.1)  t_h_ = -0.60 (-0.67) | P_e_ = 1.4×10^-3^ (1.5×10^-3^)  P_h_ = 2.1×10^-5^ (2.7×10^-5^) | μ_e_ = 1.9×10^-6^ (1.9×10^-6^)  μ_h_ = 4.7×10^-10^ (5.8×10^-9^) |
| 5 | 12.1 | 2 | Δε_e_ = -30 (-30)  Δε_h_ = -29 (-29) | t_e_ = -2.0 (-2.1)  t_h_ = -0.60 (-0.67) | P_e_ = 4.4×10^-3^ (4.6×10^-3^)  P_h_ = 6.6×10^-5^ (8.4×10^-5^) | μ_e_ = 2.0×10^-6^ (2.0×10^-6^)  μ_h_ = 4.7×10^-10^ (5.7×10^-9^) |
| 6 | 12.3 | 2 | Δε_e_ = -7.6 (-11)  Δε_h_ = -6.6 (-9.5) | **t_e_ = 13 (13)**  t_h_ = 12 (-12) | **P_e_ = 0.13 (0.13)**  P_h_ = 1.8×10^-2^ (1.9×10^-2^) | **μ_e_ = 1.7×10^-2^ (1.7×10^-2^)**  μ_h_ = 3.5×10^-4^ (3.0×10^-4^) |
| 7 | 12.3 | 2 | Δε_e_ = 7.6 (11)  Δε_h_ = 6.6 (9.5) | t_e_ = 13 (-13)  t_h_ = -12 (-12) | P_e_ = 9.4×10^-2^ (8.9×10^-2^)  P_h_ = 1.4×10^-2^ (1.3×10^-2^) | μ_e_ = 9.6×10^-3^ (7.5×10^-3^)  μ_h_ = 2.1×10^-4^ (1.5×10^-4^) |

**Table S5.** DFT calculated transport properties for p-Br DPP broken down into hopping pathways. P is the relative probability of hopping along the i^th^ pathway. t is the transfer integral, Δε is the site energy difference, m is the multiplicity, i.e. the number of symmetry equivalent pathways, and μ is the contribution to the mobility from that pathway. Dominant pathways are shown in bold. Results using the B3LYP functional are shown first, followed by the PW6B95 result in parentheses.

| Path | d (Å) | m | Δε (meV) | t (meV) | P | μ (cm^2^/Vs) |
| --- | --- | --- | --- | --- | --- | --- |
| 1 | 5.6 | 2 | Δε_e_ = -2.5×10^-2^ (-2.4×10^-2^)  Δε_h_ = 2.2×10^-3^ ( 1.1×10^-2^) | **t_e_ = 31 (-31)**  **t_h_ = -21 (-20)** | **P_e_ = 0.47 (0.47)**  **P_h_ = 0.33 (0.32)** | **μ_e_ = 6.8×10^-2^ (5.8×10^-2^)**  **μ_h_ = 4.2×10^-4^ (2.8×10^-4^)** |
| 2 | 5.6 | 2 | Δε_e_ = 2.9×10^-2^ (4.0×10^-2^)  Δε_h_ = 1.4×10^-2^ (1.9×10^-2^) | **t_e_ = -21 (-21)**  **t_h_ = 107 (104)** | **P_e_ = 0.22 (.22)**  **P_h_ = 0.84 (0.84)** | **μ_e_ = 1.4×10^-2^ (1.2×10^-2^)**  **μ_h_ = 0.28 (0.19)** |
| 3 | 7.3 | 2 | Δε_e_ = 1.1×10^-2^ (9.9×10^-3^)  Δε_h_ = 1.3×10^-2^ (1.4×10^-2^) | **t_e_ = -23 (-23)**  **t_h_ = 40 (-40)** | **P_e_ = 0.26 (0.26)**  **P_h_ = 0.12 (0.13)** | **μ_e_ = 3.4×10^-2^ (3.1×10^-2^)**  **μ_h_ = 9.3×10^-3^ (7.2×10^-3^)** |
| 4 | 12.0 | 2 | Δε_e_ = -4.1×10^-4^ (-2.2×10^-3^)  Δε_h_ = -2.2×10^-2^ (-3.0×10^-2^) | t_e_ = -0.61 (-0.65)  t_h_ = -0.08 (9.0×10^-2^) | P_e_ = 1.5×10^-4^ (1.7×10^-4^)  P_h_ = 2.8×10^-6^ (3.3×10^-6^) | μ_e_ = 4.6×10^-8^ (5.2×10^-8^)  μ_h_ = 4.9×10^-13^ (4.9×10^-13^) |
| 5 | 12.7 | 2 | Δε_e_ = 20 (18)  Δε_h_ = 19 (17) | t_e_ = 8.8 (9.0)  t_h_ = 6.9 (-7.1) | P_e_ = 2.6 ×10^-2^ (2.8×10^-2^)  P_h_ = 2.4×10^-3^ (2.8 ×10^-3^ ) | μ_e_ = 1.0×10^-3^ (1.0×10^-3^)  μ_h_ = 1.2×10^-5^ (1.1×10^-5^) |
| 6 | 12.7 | 2 | Δε_e_ = 20 (18)  Δε_h_ = 19 (17) | t_e_ = 8.8 (9.0)  t_h_ = 6.9 (7.1) | P_e_ = 2.6 ×10^-2^ (2.8×10^-2^)  P_h_ = 2.4×10^-3^ (2.8 ×10^-3^ ) | μ_e_ = 1.0×10^-3^ (1.0×10^-3^)  μ_h_ = 1.2×10^-5^ (1.1×10^-5^) |
| 7 | 13.0 | 2 | Δε_e_ = 3.5×10^-3^ (3.1×10^-3^)  Δε_h_ = -7.7×10^-3^ (-1.1×10^-2^) | t_e_ = -0.52 (-0.55)  t_h_ = -0.35 (-0.34) | P_e_ = 2.2×10^-4^ (1.2×10^-4^)  P_h_ = 4.8×10^-5^ (4.5×10^-5^) | μ_e_ = 1.1×10^-8^ (1.3×10^-8^)  μ_h_ = 4.5×10^-10^ (2.8×10^-10^) |

**Table S6.** Comparison of internal reorganization energies (λ) for DPP, p-Cl DPP, and p-Br DPP calculated with the B3LYP and PW6B95 functionals using def2-SVPD and def2-TZVP basis sets. *** indicates a calculation that failed to converge.

| Material | λ (meV): def2-SVPD basis set | λ (meV): def2-TZVP basis set |
| --- | --- | --- |
| DPP | λ_e_ = 184 (241)  λ_h_ = 317 (353) | λ_e_ = 192 (210)  λ_h_ = 331 (365) |
| p-Cl DPP | λ_e_ = 182 (192)  λ_h_ = 328 (354) | λ_e_ = 203 (***)  λ_h_ = 348 (376) |
| p-Br DPP | λ_e_ = 175 (186)  λ_h_ = 316 (344) | λ_e_ = 181 (192)  λ_h_ = 330 (360) |


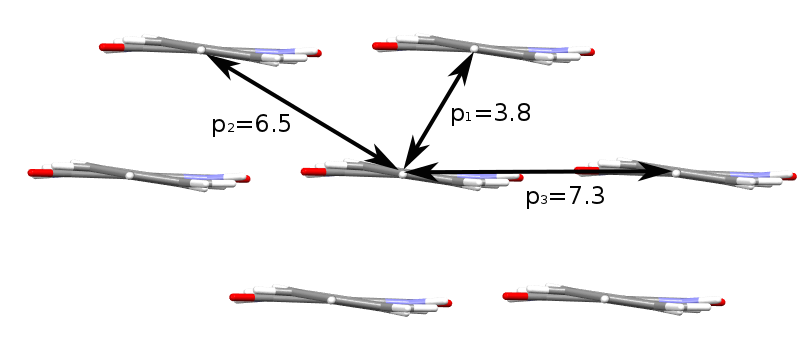


**Figure S2.** Transport pathways in DPP. Both p-Cl DPP and p-Br DPP have similar transport planes, but the relative displacement between the layers is shifted, causing path 2 to become shorter and path 1 to become longer.


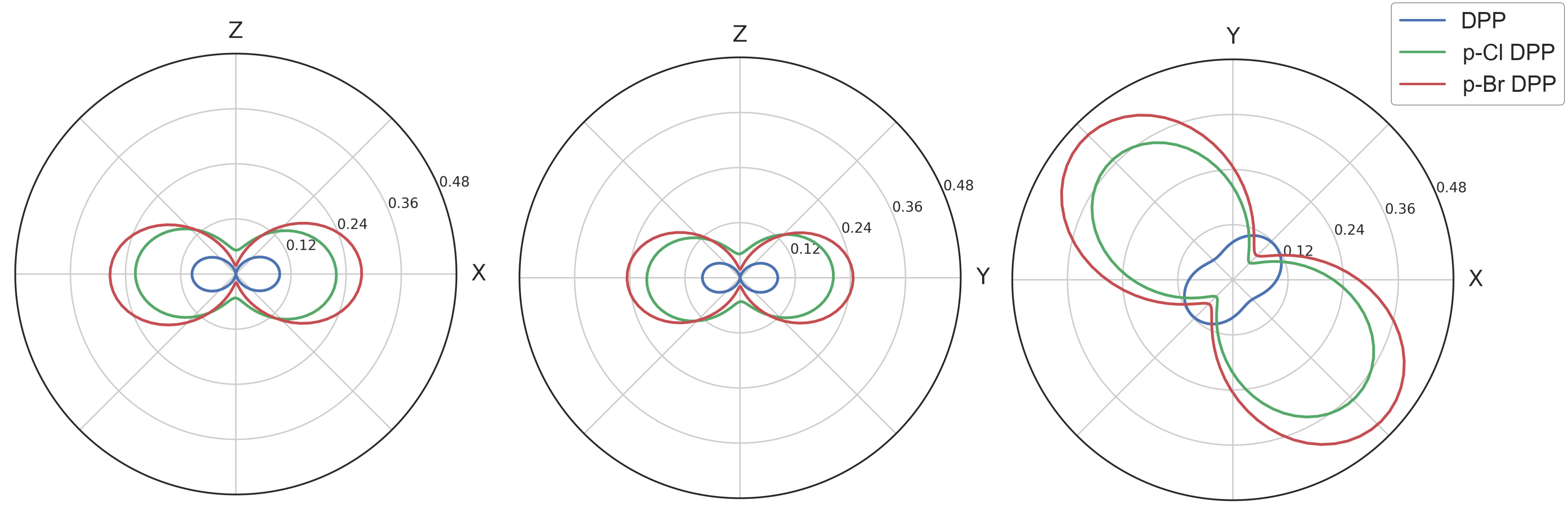


**Figure S3.** Anisotropic hole mobility $\mu_{h}\left( \theta,\phi\right)$ displayed in the XY, XZ, and YZ planes. $\mu\left( \theta,\phi\right)$ is computed from a 1D angularly projected diffusion constant computed by averaging over random walk simulations using the PW6B95 transfer integrals and charge transfer rates. The X-axis is aligned with the H-bond (p_3_ transport pathway in **Figure S2**) and the Y-axis is perpendicular to the molecular backbone in the direction of the π-π interactions (p_1_ and p_2_ transport pathways). The Z-axis is mutually perpendicular to this H-bond/π-interaction plane and corresponds to head-to-tail molecular contacts (out of the page in **Figure S2**).

**
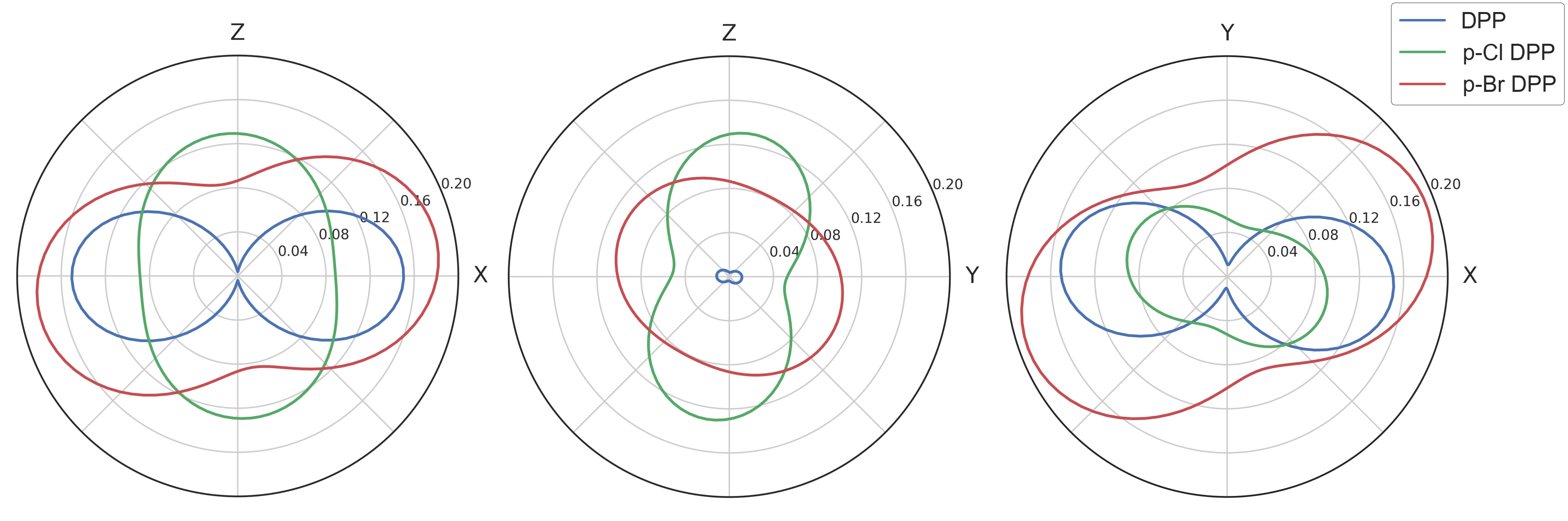
**

**Figure S4.** Anisotropic electron mobility $\mu_{e}\left( \theta,\phi\right)$ displayed in the XY, XZ, and YZ planes. $\mu\left( \theta,\phi\right)$ is computed from a 1D angularly projected diffusion constant computed by averaging over random walk simulations using the PW6B95 transfer integrals and charge transfer rates. The X-axis is aligned with the H-bond (p_3_ transport pathway in **Figure S2**) and the Y-axis is perpendicular to the molecular backbone in the direction of the π-π interactions (p_1_ and p_2_ transport pathways). The Z-axis is mutually perpendicular to this H-bond/π-interaction plane and corresponds to head-to-tail molecular contacts (out of the page in **Figure S2**).

**
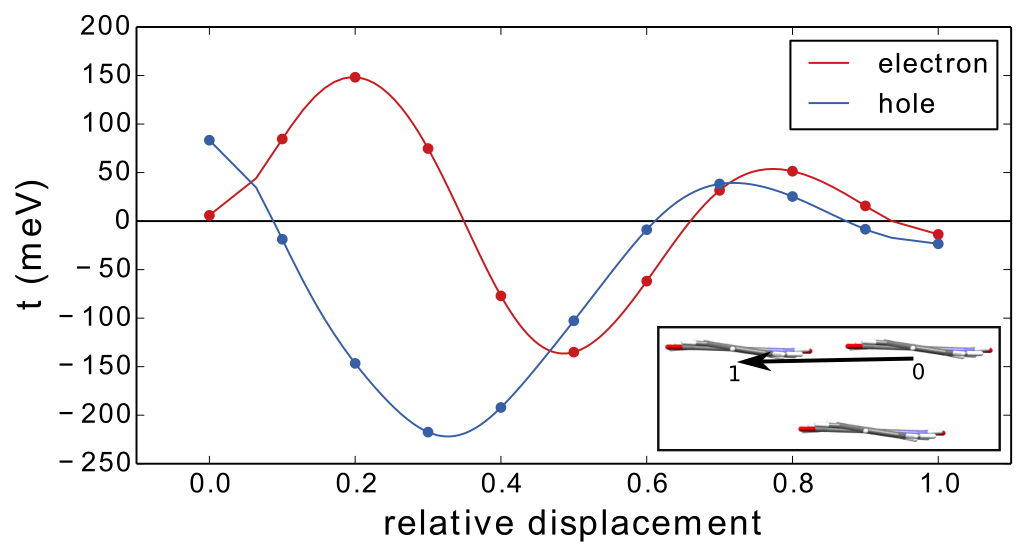
**

**Figure S5.** Translating the molecules in the first hopping pathway of DPP relative to one another along the direction shown in the inset (where 0 corresponds to p_1_ and 1 corresponds to p_2_ of the experimental structure) results in a modulation of the charge transfer integral. The 11 nodes corresponding to DFT calculations (B3LYP-D3BJ/def2-TZVP) have been interpolated with a Lagrange polynomial. The transfer integrals initially increase as the overlap in the frontier orbitals is increased by the shift toward cofacial stacking of the dimer (relative displacement ~0.25).


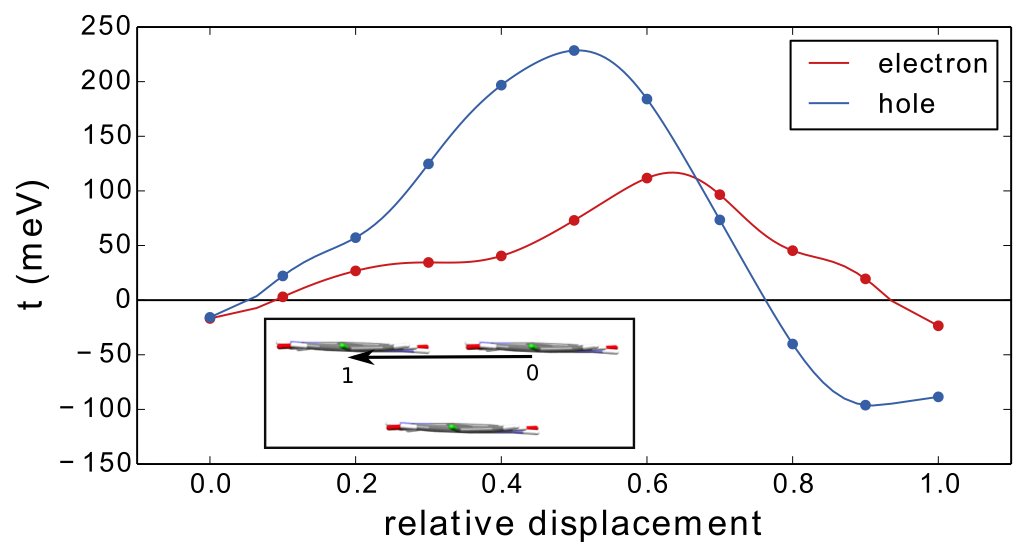


**Figure S6.** Translating the molecules in the first hopping pathway of p-Cl DPP relative to one another along the direction shown in the inset (where 0 corresponds to p_1_ and 1 corresponds to p_2_ of the experimental structure) results in a modulation of the charge transfer integral. The 11 nodes corresponding to DFT calculations (B3LYP-D3BJ/def2-TZVP) have been interpolated with a Lagrange polynomial. The transfer integrals initially increase as the overlap in the frontier orbitals is increased by the shift toward cofacial stacking of the dimer (relative displacement ~0.4).


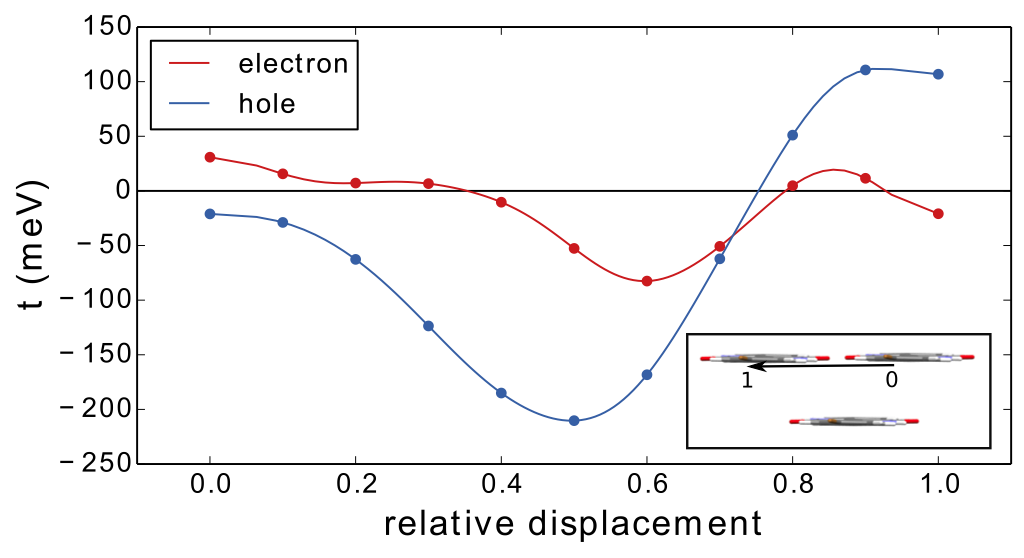


**Figure S7.** Translating the molecules in the first hopping pathway of p-Br DPP relative to one another along the direction shown in the inset (where 0 corresponds to p_1_ and 1 corresponds to p_2_ of the experimental structure) results in a modulation of the charge transfer integral. The 11 nodes corresponding to DFT calculations (B3LYP-D3BJ/def2-TZVP) have been interpolated with a Lagrange polynomial. The transfer integrals initially increase as the overlap in the frontier orbitals is increased by the shift toward cofacial stacking of the dimer (relative displacement ~0.4).
